# Relational Autonomy, Dementia, and AI-Based Care Robots: Ethical Aspects of Using Machines to Care for People with Dementia

**DOI:** 10.1007/s41649-025-00363-3

**Published:** 2025-12-09

**Authors:** Mario Kropf

**Affiliations:** Institute of Moral Theology, Faculty of Catholic Theology, University of Graz, Graz, Austria

**Keywords:** Care robots, Relational autonomy, Dementia, AI Ethics, Artificial intelligence, Robot companions

## Abstract

In the care sector, professionals face numerous challenges, for example due to a lack of resources, overburdened wards, or stressful situations with patients. In order to counter these and other stress factors, technical means have been increasingly assigned for some years, including care robots. These machines are intended to counteract staff shortages, relieve the burden on nurses, or generally consider the technical component of care. Numerous authors have dealt with ethical aspects surrounding these machines. However, the thematization of people with dementia and the associated question of relational autonomy through the use of AI-based care robots has been neglected so far. To this end, the first step is to consider dementia with reference to relational autonomy. These people are dependent on help, particularly due to the physical and mental impairments that become increasingly apparent in the course of dementia. In this context, a relational understanding of autonomy seems useful. In a second step, AI-based care robots and their current capabilities are presented. In the third step, ethical challenges and opportunities that arise from the integration of these machines for people with dementia are presented. To this end, the notion of relational autonomy is used on the one hand, and on the other hand, special reference is made to (1) a new interaction, (2) mobility, and (3) communication. It will be shown that relational aspects of autonomy can be realized to a certain extent by these robot companions, thus contributing to the care for people with dementia.

## Introduction

Caring for elderly, sick, or otherwise dependent people is challenging and can push caregivers to their limits. Due to the current shortage of nursing staff in the healthcare sector, technical aids are increasingly being considered. This also includes care robots, which allow new forms of interaction, can relieve the human staff or be perceived as support (Vandemeulebroucke et al. 2021). This article is about an ethical assessment of care robots with regard to people with dementia and relational autonomy. These people lose the ability to live independently in the course of the disease and are increasingly dependent on help (Bender 2018). This dependency results in a socially relevant problem because it can affect anyone, and thus, support is necessary. Within individualistic concepts of autonomy, these people increasingly appear heteronomous, which, however, may be avoided by a relational idea of autonomy. Through relational autonomy, a person’s preferences and interests can still be considered, for example, even if they are not communicable themselves. Such an approach seems plausible for people with dementia, but it is important to clarify whether robots can also provide the support in question. Although some authors (Ienca et al. 2016; Sharkey and Sharkey 2012; Draper and Sorell 2017; Sorell and Draper 2014; Chita-Tegmark and Scheutz 2021; O’Brolcháin 2019) have already addressed ethical aspects of care robots, they mostly evaluate *pet-like* robots or the robotic seal Paro. Socially assistive robots (SARs) with more extensive functions are increasingly being discussed, but not yet sufficiently. In a first step, dementia is linked to a form of relational autonomy. This approach seems reasonable because special attention is paid to the social dimension of an autonomous way of life. Based on these considerations, the second step is about AI-based care robots. It is shown what the technical devices can currently do and what not, in order to assess their relevance for the care of people with dementia. The third step is an ethical analysis based on the previous considerations, which illuminates three aspects in particular. Attention is paid to (1) the new form of (human–robot) interaction, (2) a possible mobilization, and (3) communication. This approach results from the discussed literature on the one hand and, on the other, seems crucial for the *technical* care of persons with dementia. This article addresses the following research question: How can the use of AI-based care robots help to strengthen the relational autonomy of people with dementia?

## Dementia and Relational Autonomy

This section addresses the connection between dementia and a notion of relational autonomy and also shows the advantages of this relational approach over individualistic notions of autonomy. Dementia is a clinical syndrome characterized by, among other things, reduced memory function, worsening physical condition, psychiatric abnormalities, or impairments in everyday life (Bender 2018). Due to demographic change and in agreement with Alzheimer’s Disease International (2025), it can be assumed that the number of people with dementia will continue to rise. While approximately 55 million people worldwide currently live with dementia, scientific estimations suggest that these numbers will more than double by 2050 (Alzheimer’s Disease International 2025). Dementia thus affects everyone, either directly or indirectly. For individuals with dementia, there is a physical and psychological dependency, and they rely on other people (Low and Purwaningrum 2020). This *reliance on others* makes it essential that these people are cared for and looked after, which, in addition to professional care and, for example, family caregivers, also brings the care robots discussed in this article into focus. Dementia seems to cause a lot of insecurity, fear and worry among the population (Herrmann et al. 2018), which could possibly be intensified by the use of AI-based robots—or reduced. What follows from the above is the necessity to provide care for people with dementia. It seems plausible to claim that this care should correspond to the wishes and preferences of the person in question. For this very reason, an approach based on relational autonomy seems reasonable, because personal ideas of a meaningful life can also be conveyed by others.

However, it should be noted that there are many different concepts of relational autonomy (Gómez-Vírseda et al. 2019). This article is specifically based on the perspectives^1^ of Mackenzie, Stoljar, Anderson, and Christman, although other contemporary authors are also considered. Roughly speaking, several assumptions can be derived from the works mentioned that correspond better to a substantive approach: A person can *firstly* be seen as a socially embedded actor in the context of relational autonomy (Christman 2004; Mackenzie and Stoljar 2000). *Secondly*, autonomous decisions can be influenced by social relationships (causally), and at the same time cannot be explained without them (constitutively), because we define ourselves as social beings (Mackenzie 2019a; Anderson 2013). *Thirdly*, this seems to be associated with the necessity of enabling suitable conditions for an individual (and autonomous) way of life (Christman 2014; Mackenzie 2019b). If, in this understanding, relational autonomy depends not only on individual but also on social aspects, then it is important to make the social available to every individual. Based on these assumptions, it can be stated that excessive demands regarding relational autonomy should be avoided. This could lead to problems regarding people with dementia and to an idea of individualistic autonomy. Relational autonomy can pay more attention to these persons, as the *individual loss* could be compensated by social support, as will be shown below.

Focusing on this understanding of relational autonomy not only makes it possible to avoid a concept of freedom that is often understood as being too individualistic—and *atomistic* (Anderson 2013, 62), and therefore not corresponding to the current reality of life—but also suggests that a relational dimension^2^ is particularly fruitful for persons with dementia. Due to the cognitive limitations described above, which become more and more pronounced as the disease progresses, and with them the diminishing ability to make autonomous decisions, the environment of the person affected becomes crucial. Considering social networks, important people and shared values usually characterizes an idea of relational autonomy (Christman 2014; Dove et al. 2017; Christman and Anderson 2005; Mackenzie 2019a). In this context, an autonomous decision, for example by a person with dementia, cannot be thought of without their friends, family, loved ones or even influential groups, because it is precisely these associated aspects that make up the person at their core (Anderson 2013). If a nurse has internalized the values of compassion or truth in the course of her life and these shape her own way of life and action, then the people close to her will also have influenced these values—or will be equally influenced by these values and her attitude.

The way we as persons are perceived by others, perceive them, and want to be perceived, in a sense shapes this notion of relational autonomy. By contrast, an exclusively individualistic conception of autonomy would have to ignore these relations, the people and values behind them, to a certain extent (Menke 2010). Otherwise, external influence—on subject-related autonomy—by these very people seems unavoidable, which may constitute a restriction of freedom (Dove et al. 2017). By comparison, a relational conception of autonomy can do justice to these diverse influences because it “places the individual in a socially embedded *network* of others” (Dove et al. 2017, 153). Regarding people with dementia, it becomes clear that autonomy is often called into question even at the beginning of the disease. Conventional individualistic views of autonomy are based, for example, on a moral universalizability (Horn et al. 2015), a stage theory (Frankfurt 1988) or the general idea that the person in question identifies with their own ideas, desires and goals—and then acts on them (Buchs 2018). For people with dementia, this requirement not only seems to be *overly demanding*, but also becomes increasingly difficult to meet as the disease progresses.

There are memory lapses, connections are no longer recognizable, one’s own desires may change, one’s own self can rearrange itself, and in general, rational decisions seem more difficult to implement (Trahan et al. 2011; Ebert et al. 2020; Bender 2018). Apart from the general difficulty of determining (individual) autonomy in persons with dementia in various phases of the disease, relational autonomy offers several advantages. From the previous considerations, and also with reference to the works of Mackenzie, Stoljar, Anderson, and Christman, certain conclusions can be drawn that seem useful for this article. Friends or close family members can (A) help to determine the wishes of the person with dementia in medically important decisions, which may no longer be ascertainable,^3^ and act accordingly (Specker Sullivan and Niker 2018; Le et al. 2024). Based on their relationship to the person concerned, they know the specific preferences, dislikes, beliefs, and values that have shaped that person up to that point—even if the associated self (Oshana 2005) can no longer be expressed at a particular point in time. In this context, it is entirely plausible that (B) considering the aforementioned indicators also contributes to respecting and promoting the autonomy of the person with dementia (Christman 2014). If the relatives or other trusted persons not only act in the best interest—or their own interest—but actually draw on the earlier self and associated aspects for a decision, this also preserves the (relational) autonomy of the person concerned.

Personal ideas of a meaningful life can thus be conveyed by the individuals close to the person with dementia, not least because these values are in many cases also relevant to them. Anderson emphasizes that a dialogical approach and the associated exchange are essential for relational autonomy (Anderson 2013). In the context of dementia, the need to enter into dialog with others quickly seems to reach its limits—because communicative abilities disappear. However, this does not mean that autonomy is impossible, precisely because the people who are in the *network* of the person with dementia can also conduct this dialogue as surrogates. Oshana’s comments are to be understood in a similar way when relational autonomy describes a *predominantly remaining true to oneself* (Oshana 2005). This leads to the last important component in the context of dementia and relational autonomy, because (C) the social environment and the individuals within it must take responsibility (Specker Sullivan and Niker 2018). It is not only about (A) identifying personal ideas about life—of the person with dementia—and (B) maintaining their relational autonomy, but also about (C) taking responsibility for being able to justify the respective decision.

Anderson even says that without this fundamental willingness to be able to give reasons for one’s own actions to other people, any attitudes and associated decisions are not only outside the spheres of relational but also of individual autonomy (Anderson 2013). Christman (2014, 380) presents a similar idea because ”we reflect on our individual situations in a dialog with others, both present to us and imaginatively“. What is needed in the context of the aforementioned perspectives are certain people who care about the person with dementia, to some extent set aside their own goals, and are responsible (Christman 2014; Dove et al. 2017). They ideally take on the important conversations, for example in the hospital or a care facility, maintain (relational) autonomy and can justify their decisions (Anderson 2013). While people with dementia can ultimately be described as non-autonomous in an individualistic model, a relational understanding—in which others play a significant role—provides a simpler, more practical and more realistic explanation of how autonomy can be conceived. However, it remains to be determined whether this form of relational support can also be provided by care robots.

## AI-Based Care Robots

After describing dementia and a perspective on relational autonomy, the performance of current care robots is considered. This enables the subsequent ethical analysis and reveals the relevance for people with dementia and their relational autonomy. Care robots are physically embodied artificial agents that display certain characteristics so that they can be perceived as social entities by people in need of care, enable interaction and communicate verbally and non-verbally (Naneva et al. 2020; Soljacic et al. 2024). They are developed specifically for vulnerable groups (e.g. the elderly, children, and people with dementia) and fulfill a variety of tasks, such as administering medication, monitoring health, enabling social contact, or accompanying patients (Chan 2021). In most cases, the aim is to relieve staff—or to respond to the current shortage of specialist nursing staff in many institutions—to improve the quality of care or to exploit technical options (Vandemeulebroucke et al. 2021; Chu et al. 2017; Hung et al. 2022). This article deals with socially assistive robots (SARs), which, in addition to purely technical assistive work, are also designed to fulfill the social component of care (Chu et al. 2017; Obayashi et al. 2020). They interact with people in need of care, for example by bringing medication or checking vital functions, and also contribute to social engagement. The robotic seal Paro^4^ or other *companion robots* may also fall into this category. Although such companions can also promote social behavior or lift the spirits, their technical capabilities and, in particular, the design of the human–robot interaction are more limited.

For this reason—the technical-practical potential—and especially due to the fact that more advanced care robots for the care of people with dementia have not been sufficiently considered so far, the focus is on those machines. Although (ethical) literature on the use of SARs already exists, studies on the use of Paro are comparatively more abundant. Furthermore, the focus on relational autonomy, SARs, and people with dementia represents a new approach. Such machines offer verbal interaction possibilities and can move (at least partially) autonomously in a predefined environment. It also seems to make a difference whether, for example, a care robot reads a story from a book or the people being cared for read the same story on a tablet (Zuschnegg et al. 2022). The physical presence of the machine and the communication can be decisive in why many people in such scenarios decide against the mere offer (tablet) and rather for the kind (care robot) of mediation—that is, the technical presentation of the corresponding offer (Yuan et al. 2022; Ghafurian et al 2021). According to Sharkey and Sharkey (2012), robots can be assigned to (1) assistance, (2) health monitoring, or (3) companion care —the machines addressed in this article theoretically fulfill the requirements for all three categories.

Examples include PR2, NAO, and the Kompai robot. Their many functions include administering medication, playing videos, monitoring patient-specific data or picking up and delivering certain items. In general, they are able to interact with the people being cared for through verbal or visual output functions. Comparable robots such as Pepper, Lio, or Care-O-Bot are also suitable for this purpose and can also be classified as SARs. The robot MARIO is well suited for people with dementia because it promotes social contact with friends or family members, makes games, music or even news available (Mannion et al. 2020). RobAlz is a care robot that was developed explicitly for people with dementia. It is designed to delay cognitive decline, point out activities and appointments, or even trigger an alarm (iTMunch 2019). Certain *features*, such as the ability to communicate verbally and to move independently, appear to be important for the care of people with dementia (Zuschnegg et al. 2022; Ghafurian et al. 2021). However, the question arises as to what extent SARs have an advantage over *pet-like* robots or *companion* robots (Roger et al. 2012). Is verbal or visual communication necessary between people with dementia and care robots? Does it make sense for technical aids to encourage these people to exercise?

It should be noted that the following considerations are limited to the early stages of dementia. In particular, at this stage, affected people can still interact for the most part, i.e., communicate or perform certain physical activities, but also need special social care (Tampi and Jeste 2022). In later phases, due to the progressive course of dementia, complete dependence on other people usually is the case. In these phases, according to the current state of care robots, it makes little sense to assign machines that require a certain degree of interaction potential on the part of the other person (Ienca et al. 2016). Although they encourage mobility, for example, in most cases, they cannot dress or get a person out of bed on their own. These robots are unsuitable for completely taking over personal hygiene. Furthermore, Hung et al. (2022, 4) are right in saying that a lot of work is needed “to motivate people learn about what the robots do and how to use them”. That being said, the aim is to determine what preventive or prophylactic skills care robots can contribute to the caring relationship with people with dementia—and thus also strengthen relational autonomy.

The decisive factor is to live as independently as possible with the support of technical aids, to promote well-being, and to emphasize the wishes and specific needs of people with dementia—which, based on what has been said so far, can be achieved by focusing on relational autonomy. In order to do justice to this disease and, more importantly, to the individuals affected by it, the following section will refer to the challenges that typically affect this group of people. These include the fact that (1) even at the beginning of the disease, people with dementia repeatedly forget people they actually know, (2) their ability to communicate and interact may decrease drastically, (3) their physical condition suffers due to a lack of movement, and (4) as a result of the aspects mentioned, their social interaction, which is essential for their well-being, is diminishing. Based on the above and according to the assumptions outlined at the beginning of chapter two, the relevance of relational autonomy for the present article can be summarized as follows: *Firstly,* relational autonomy recognizes a person as a socially embedded agent, which means that AI-based care robots can contribute to this social life (e.g., ANT and footnote 6). *Secondly,* relational autonomy not only recognizes social (external) influences for the person concerned but also considers such influences to be valuable in at least some respects (Walter and Ross 2013). And *thirdly,* relational autonomy seems particularly fruitful and reasonable for people with dementia. The special needs of this group of people also justify special recognition regarding the self, personal values or preferences, even if they cannot be fully conveyed by the person themselves (Kropf 2024). Therefore, appropriate conditions must be created for a good (and autonomous) life that includes others and their contributions. A detailed analysis of these considerations and ethical aspects will follow in the next sections.

## Ethical Analysis

After the presentation of SARs, relational autonomy, and dementia, ethically relevant dimensions associated with robots must be addressed. Even though the use of AI-based care robots for people in need of care has been widely discussed, SARs related to relational autonomy and dementia are yet to be addressed. In the further course of this section, three aspects (interaction, communication, and mobility) will be discussed in more detail, which are linked to relational autonomy and require special attention for individuals with dementia.

### New Interaction and Cognitive Activation

While we usually deal with familiar, trusted, and predictable behavior in human–human interaction, this is no longer the case when humans and robots interact. The experience embedded in the memory of many people can no longer be used to assess or anticipate the actions and decisions of AI-based robots, precisely because in very many cases there is no (or not yet) any relevant experience (Persson et al. 2024). When people with dementia are cared for in a residential facility—or at home—it is often human nursing staff who are initially responsible for their well-being. Only gradually are technical solutions beginning to offer a way to establish this kind of experience. The robot most commonly used in the care of people with dementia is the robotic seal Paro, as mentioned above, because many studies (Honekamp et al. 2019; Roger et al. 2012; Moyle et al. 2019; Koh et al. 2023) confirm its positive effect *on* and *for* those affected. By contrast, the use (and scientific discussion) of SARs has only increased in recent years.

For people with dementia, this *new form of* interaction with a robot has a positive and valuable consequence, namely, that it puts them in a comparable position to all other people. While the cognitive performance of people with dementia continues to deteriorate due to the effects of the disease, and (short-term) memory is also affected (Nebel et al. 2018; Leung et al. 2020; Tamura et al. 2004), the technical relationship can be (cognitively) stimulating for people with dementia, making forgetfulness obsolete to a certain extent and contributing to a sense of well-being. Compared to people who do not have dementia, the (theoretical) experience of interacting with robot companions in the care sector would most likely have disappeared again, which may put people with dementia at a disadvantage.^5^ However, since AI-based robots represent a completely new intervention option for the care of people requiring care, cognitively *healthy* people will not have an advantage—that is, an already established experience. Furthermore, the forgetfulness described suggests that people with dementia may repeatedly perceive this unusual and innovative relationship^6^ with a robot as enriching.

When human caregivers reach their limits, have no time to help, or their approach is unsuccessful, technical assistants are a valuable alternative. By interacting with Pepper or RobAlz, participating in games that may be suggested, or simply paying attention, people with dementia can be cognitively activated (Pfadenhauer and Dukat 2015; Yuan et al. 2022; Chu et al. 2017). This does not mean that the technical form of interaction can stop the loss of cognitive capacity, but at least some prevention is possible to some extent—as studies (Zuschnegg et al. 2022; Law et al. 2019; Obayashi et al. 2020) suggest. This specific-technical relationship represents an option for people with dementia to repeatedly benefit from the *new kind of* interaction, so to speak. AI-based care robots, for example, help to maintain relational autonomy (B) by counteracting cognitive decline through interaction on the one hand, and on the other hand, by offering a general option for taking people’s concerns into account (Christman 2014). In this way, the social embeddedness of people can be strengthened, even when robots are part of that social structure.

If these machines can counteract the cognitive decline of people with dementia through the novel form of interaction—as the above suggests—then this option should be made available. Otherwise, this group of people would be deprived of an opportunity to maintain and promote their mental and cognitive health, which is difficult to justify. This can be compared to the norm proposed by Beauchamp and Childress (2019, 159): “Do not deprive others of the goods of life”. This does not imply that people with dementia have an obligation to use the technical aids, but rather that the provision of this option (the good) is important, although its use should be approved by the (relational) autonomy of the individual in question (Sorell and Draper 2014). Furthermore, interaction—and cognitive activation—strengthens relational autonomy *if* and then *because* people with dementia are longer able to think clearly, theoretically find it easier to communicate or express themselves. The importance of dialog and exchange has already become clear, which is also underscored by the statement of a person receiving care: “I think to remind you to exercise is very good” (Law et al. 2019, 5). This also highlights the relevance of social networks that can provide such support (Dove et al. 2017; Lewis 2019; Hunt and Ells 2011; Vanlaere and Gastmans 2011).

It can certainly be argued that this *novelty* will simply disappear over time and with more routine (Robaczewski et al. 2021). Due to the fact that people with dementia are confronted with cognitive impairments in the course of their illness, the factor of novelty seems to lose little of its relevance. It is possible that these people will tomorrow no longer remember the conversation they had with Lio today. Furthermore, it seems plausible that constant development always guarantees a certain degree of novelty (Chita-Tegmark and Scheutz 2021). A potential danger would exist particularly if the machines were frightening, scary, and uninteresting. Then, people with dementia would have to overcome themselves repeatedly, giving other people a crucial and ethically significant advantage. This means that this personally perceived barrier must be considered in order to avoid harming people with dementia (Beauchamp and Childress 2019). Apart from that, it is important, *firstly,* to avoid a corresponding appearance (or the conceivable aversion) before integration into a care facility and, *secondly,* study results (Law et al. 2019; Yuan et al. 2022; Chu et al. 2017; Nakamura and Umemuro 2022) point in the opposite direction—according to which people with dementia are not (generally) afraid of technical aids.

This *new form of* interaction should, however, also be viewed critically, because it can also be strange, uncertain, unfamiliar and possibly threatening. With the onset of dementia, the ability to remember recent events fades, but especially in the early stages, the long-term memory remains intact (Leung et al. 2020). It is therefore conceivable that individuals can remember experiences long past and at the same time forget whether they have already had lunch. This brings another advantage to light, because SARs do not play a role in the long-term memory of persons with dementia. If memories are free of technical helpers, then no bad experiences with these machines can be found in the memory either. Authors (Misztal 2012; Tamura et al. 2004; Roger et al. 2012) emphasize that long-forgotten experiences, early childhood traumas, or even frightening experiences contribute to avoiding comparable scenarios in the future for precisely this reason. However, a person requiring care will not yet know how Lio, for example, reads a story aloud or how Care-O-bot walks with them through the corridors. In addition, the appearance of the robot plays an important role when it comes to triggering or avoiding possibly deeply rooted experiences (Dosso et al. 2022; Nakamura and Umemuro 2022). This circumstance must be considered during development and design, but it should be noted that (relationally) autonomous decisions can be positively influenced and constituted by these social relationships (possibly also with robots).

Based on the considerations so far, the *new form of* human–robot interaction can be useful for individuals with dementia, essential for their own well-being, and preventive or beneficial for cognitive performance. The currently limited experience of many affected persons basically suggests that people with dementia have no disadvantages—but rather an advantage—compared to cognitively *healthy* people, and that no frightening experiences influence the future interaction with robots. A relational form of autonomy can be (B) preserved if the robot companions consider the wishes of those affected. AI-based care robots can stimulate cognitive activity, involve people with dementia in memory games, or even perform certain tasks for them (Law et al. 2019; Obayashi et al. 2020). This reveals a relational dimension, or in other words: “interaction with vulnerable others can facilitate the development of their autonomy” (Christman 2014, 381). In addition to this development, the main focus here is on preserving the autonomy of people with dementia and expressing it in relational terms.

### Communication and Speech

Communication and exchange are crucial in the context of relational autonomy. At the same time, however, dementia increasingly limits the communicative abilities of those affected (Roger et al. 2012; Williams et al. 2009; Banovic et al. 2018). These individuals are less and less able to express themselves and their personal needs due to the progressive nature of the disease. The use of AI-based care robots could be an option to delay these communicative losses in the early stages of the disease and, in later stages, to take over some of the communication-related tasks for people with dementia. In order to identify the wishes, preferences, and general fears of those affected, modern care robots are equipped with specific technology. Inputs from the environment are sensed and lead to an associated output, such as a movement (Pfadenhauer and Dukat 2015). The processing usually takes place in steps that are difficult to understand.^7^ Nao and Pepper, for example, would pay attention to gestures, facial expressions, or even the explicitly expressed wishes of a person in need of care (Chu et al. 2017; Robaczewski et al. 2021). However, it should be noted that the detection of non-verbal forms of expression currently still poses major challenges for robots and can therefore only be achieved by the machine with some limitations (Tanioka et al. 2021). What is already causing problems for human caregivers in relation to people with dementia is much more difficult for machines. In addition, studies illustrate the importance of adequate communication insofar as robots communicate loudly enough, not too fast, and appropriately for the target group (Law et al. 2019; Yuan et al. 2022).

Apart from that, it seems possible to strengthen the relational autonomy of people with dementia using AI-based care robots if, in a first step, the respective interests and desires of the individual *in question*^8^ are identified (A) as such. This *technical* option can be seen as “a view that puts those giving aid to a person […] into the position of helping to establish her autonomy as well as recognizing and respecting it” (Christman 2014, 376). The relational character is crucial in this context, whereby both humans and machines contribute to considering the autonomous decisions of a person with dementia (Specker Sullivan and Niker 2018). This requires the determination of preferences, which are then communicated to a human nurse, for example. Even if this determination can be implemented equally and possibly even better by human actors, this technical approach represents a novel option. Nurses do not always have the time, may be distracted by other tasks, or may lack the motivation to care for the person in question (Persson et al. 2024). As socially embedded actors, people can benefit from robots if they enable additional options for action and create suitable conditions for an individual (and autonomous) life. In these and similar situations, care robots are useful and can help to care for and consider the person with dementia.

In a further step, machines can use the identified preferences to preserve (B) the autonomy of people with dementia in a relational understanding. This preservation can be associated with a specific action, which Zuschnegg et al. (2022, 1273) convey in a similar way: “SARs could offer support with making phone calls”. In this case, it is conceivable that the robot companion could act as a mediator for the respective message or could communicate directly with the person with dementia. In the first scenario, NAO would alert the nurse to the fact that the person requiring care needs to use the bathroom, which (A) highlights certain needs. Whether or not relational autonomy is maintained then lies, to a certain extent, in the hands of the human actor. However, if NAO independently accompanies the person with dementia to the toilet, (B) *relational* autonomy is considered. Studies (Zuschnegg et al. 2022; Robaczewski et al. 2021) rightly point out that such accompaniment does not correspond to that of a human being. NAO or Lio cannot lead the person to be cared for by the hand or even provide physical support when walking.

That being said, it is justified to claim, based on the current state of technology, that AI-based care robots do not pursue their own (arbitrary) goals compared to their human counterparts and can generally act *more objectively*. In the context of the example described above, this means that the machine will adhere to the received inputs and the outputs (instructions) associated with the programing (Poulsen et al. 2018). A deviation out of a technical whim can be ruled out at present, which ensures a certain degree of objectivity. Authors (Zuschnegg et al. 2022; O’Brolcháin 2019) emphasize a *technical* advantage that appears to be particularly relevant for communication *by* and *with* people with dementia. The disease makes it likely that those affected will no longer remember recent experiences and will (apparently) constantly ask the same questions. For human caregivers, repetitive questions and statements can cause them to quickly stop talking or to avoid communication altogether. Tanioka et al. (2021, 2) also recognize this problem because “there are times when lapses in compassion and conscience can result in compromised patient care”. However, in doing so, they fail to consider the patient’s autonomy, which still exists in a limited form. Even if it becomes increasingly difficult to convey preferences, this does not mean that they have disappeared (Williams et al. 2009). Gómez-Vírseda et al. (2020) are right to speak of a gradual understanding when it comes to maintaining a degree of autonomy.

Robots offer a promising approach here because they always respond to repetitive questions or instructions and never tire. Caregivers also see great potential here when individuals with dementia reach a phase “where they ask the same question 100 times. Because the robot has no everyday stress, it does not matter” (Zuschnegg et al. 2022, 1274). In contrast to this explanation—with the reference to the non-existent stress—this *ability* of robots seems to be based more on the fact that they are programmable machines and not humans. Relational autonomy can therefore be strengthened by the use of these robot companions if they (A) elicit the preferences of the person concerned or (B) communicate directly. Similar conclusions can be drawn from the results of Persson et al. (2024, 1195), insofar as a robot “can stimulate the user to engage in communication with others”. In this case, the machines do not enable surrogate communication, but they do promote an exchange through their presence.

A (C) possible justification does not appear to be fully realizable for care robots. Either one takes the entirely plausible view that the concept of justification makes no sense in relation to AI-based systems because specific actions only correspond to programming and no reasons can be presented (Sweeney 2023). Or the concept of justification is also considered useful outside the sphere of human subjects. Then, it is understandable to say that Car-O-bot or Pepper *justifies itself* to a nurse for the trip in the hallway with Ms. X by saying that this is what Ms. X wants. This visual or verbal justification is not based on a consideration of different reasons but refers to the code integrated in the machine—but it still *appears* as a justification. If this second variant is correct, which is quite plausible, then relational autonomy can be strengthened by AI-based care robots in this case as well. The provision of decisive reasons—regardless of how this output arises algorithmically—will be important for people with dementia, for the nursing staff and generally for those affected. In this respect, it can be argued that robots offer an additional suitable condition for an individual (and relationally autonomous) way of life that should be provided.

### Mobility and Technical Support

The mobilization of people with dementia is important, especially due to the continuously increasing and currently unavoidable physical impairment (Blankevoort et al. 2010; Lam et al. 2018). To make the lives of these people pleasant and enjoyable for as long as possible, and to prevent a further deterioration of their situation, physical interventions are recommended (Chu et al. 2017; Ghafurian et al. 2021). However, the question arises as to what extent care robots can be meaningful in this regard? Apart from the limitations already mentioned, several advantages over PARO, for example, quickly become apparent. SARs can go for walks with people who need care or encourage them to be physically active, which is not possible with less autonomous technical aids (Pfadenhauer and Dukat 2015). This technical *capability* also makes it possible to demonstrate certain movements—or play them on the display—and thus take on the role of a (technical) mediator or trainer (Robaczewski et al. 2021; Chita-Tegmark and Scheutz 2021). In this sense, a person’s relational autonomy can be (B) strengthened by providing technical (and social) options. A plausible strategy regarding people with dementia would be to motivate them to move around by having Care-O-bot demonstrate procedures, present information or accompany them, which Sharkey and Sharkey (2012, 31) see similarly: “the robot can follow the occupant from room to room”.

In this context, potential surveillance by the data-based devices could be problematic, or at least such a perception by the people being cared for (Dosso et al. 2022; Ienca et al. 2016). Sharkey and Sharkey (2012, 32) also point out this danger because “there is a risk that monitoring could infringe on the right of privacy”. However, it should be noted that comparable surveillance also exists when a nurse walks (and talks) with the person concerned through the corridors. To see a danger here only in the use of technical assistants seems to express prejudices and fears about AI-based robots rather than a plausible argument. If robots are *technically* obliged to maintain privacy by their programing, then, given the current state of the art, it is less plausible to claim that they disclose intimate and private information than it is for human actors (Kropf 2025). Otherwise, one would have to believe that these technical tools have a will of their own, or at least that they can circumvent the safeguards built into their code at will. Furthermore, it is legitimate to ask what the alternative should be. Most people would probably not only accept a certain amount of monitoring^9^ and observation for this group of people, but also consider it reasonable and necessary, rather than granting them complete autonomy and leaving them to their own devices, so to speak. Such accompaniment (and, in the worst case, surveillance) not only indicates social influence on autonomous decisions, but can also strengthen relational autonomy. This is the case when the concerns of people with dementia can be (A) identified and (B) possibly acted upon.

Another problem concerns the limitations of technical devices in terms of mobilization. In the worst case, the care robots presented in this article, such as Pepper, NAO, Care-O-Bot, or RobAlz, cannot independently lift a person with dementia, help them out of or into bed, or carry out heavy lifting in general. In these situations, the machine is of no help. However, based on the previous discussion, this is not how they are intended to be used. Furthermore, this argument does not seem conclusive. Most authors who are critical of the use of AI-based care robots—and even proponents would most likely agree with this consideration—see the technical assistants *exclusively* as a support for human care staff, but by no means as a replacement (Thompson 2017; Ryan 2020; Chita-Tegmark and Scheutz 2021; O’Brolcháin 2019). For this reason, the demand that NAO, for example, may only be used if it can help a person with dementia out of bed without human assistance would be difficult to understand. If robots are to be used to support human care workers in a care facility, it cannot be demanded that such tasks be performed alone. Firstly, this would misuse the limited capacities of the machine for its incomprehensible exclusion, and, secondly, it would ignore the fact that the human counterpart is also unable to perform in comparable cases the tasks required of the machine.

In the example described, even a single human caregiver will struggle to help the person with dementia out of bed. In many cases, the cooperation of several individuals is necessary for this, which means that this limitation of care robots does not disappear into thin air but can be understood as an unjustified excessive demand. At least in theory, the use of AI-based care robots for the mobilization of people with dementia allows for the (A) identification and (B) maintenance of relational autonomy. This occurs when the machine considers a certain action to be important based on the expressed or non-verbally communicated preferences of the person in question (Ghafurian et al. 2021). Being accompanied through the hallway by Pepper or being shown physical exercises by Nao can, with the appropriate encouragement to move, represent a form of relational autonomy. Of course, it can also be argued that people with dementia are autonomous precisely when they freely decide to remain seated or generally against movement (Oshana 2013). This is not contested. Rather, the decisive factor is the technically provided option for mobilization in order to accommodate people with dementia and their relational autonomy. Johnston recognizes a danger when robots disregard the autonomy of those being cared for in order to avoid harm—such as falling out of a chair (Johnston 2022). In this case, too, it can be argued that absolute freedom does not appear to make sense. If a human caregiver does not react, this is referred to as nursing neglect, whereas the possible intervention of technology is perceived as heteronomy. It is much more plausible to assert that both human beings and technical entities can intervene in such a case, and that this even strengthens relational autonomy.

## Final Considerations

After considering ethical aspects that may arise from the use of AI-based care robots and the associated form of relational autonomy, the final issue to be examined is the often-feared social isolation of people with dementia. A relationship, or even social well-being, requires human closeness, at least that is the common assumption (Tampi and Jeste 2022; Dosso et al. 2022; Sharkey and Sharkey 2012; O’Brolcháin 2019). The use of AI-based care robots cannot provide human closeness because these machines are non-human entities. However, the situation is different when not only *human* affection, attention and closeness are meaningful, which Sorell and Draper (2014, 190) also emphasize, because those in need of care “can feel that they are not alone” —when interacting with a robot. O’Brolcháin (2019), on the other hand, considers this to be impossible and fears social deprivation for people with dementia as a result of care robots. Empirical results show that affected individuals can also feel comfortable with a robot and prefer it to human caregivers in some situations or with regard to some tasks (Honekamp et al. 2019; Nijssen et al. 2022; Oksanen et al. 2020; Chu et al. 2017; Nakamura and Umemuro 2022). This may also result from the fact that precisely this technical otherness or difference leads to this relationship (Christman 2014; Coeckelbergh 2012; Law et al. 2019) being perceived as something special, which a person in need of care emphasizes with regard to the interaction: “It was like you were relaxing. I thought the robot was adjusting to me, too” (Nakamura and Umemuro 2022, 1947). Perhaps it is not a matter of simply accepting interaction with a robot as a necessary evil, but of preferring it to interaction with humans (at least to some extent) precisely because one is aware that it is a technical care solution.

The above considerations regarding relational autonomy in the context of dementia reinforce the impression that AI-based care robots should also be seen as a useful option for supportive care. The existing limitations of the machines must be considered but should not obscure the existing potential. In particular, the reference to the group of people addressed in this article, their interests, and the best possible care for them, argues for intensive discussion. The presentation of a relational idea of autonomy showed that an interactive exchange is crucial and that autonomous decisions are largely shaped by the social environment. In the context of care, an overly atomistic notion of autonomy seems inappropriate, unrealistic, and ethically questionable. The essential aspects of relational autonomy relate to (A) the identification of specific life beliefs, (B) the preservation of associated convictions, and (C) the justification for a particular decision. In order to assess the relevance of AI-based care robots, (1) a new interaction and the opportunities it offers were pointed out, (2) the communication problems were discussed, and (3) the importance of mobilizing people with dementia was emphasized. By combining these three dimensions—in which the presented machines can be useful—with relational autonomy, a technical potential for people with dementia opens up that needs to be considered.

## Data Availability

Not applicable.

## Footnotes

1 These perspectives allow certain assumptions for relational autonomy to be derived, although it does not correspond to any definition. A definition of relational autonomy seems (1) impossible due to the wide range of concepts that describe, for example, procedural, substantive, causal, constitutive, strong or weak forms of autonomy. A definition is (2) not necessary for this article because it is not about a specific concept of relational autonomy per se, but rather about the applicability of this idea to AI-based care robots and people with dementia. The derived assumptions seem sufficient for this. And (3) the assumptions described here can be understood in such a way that a viable definition of relational autonomy will at least implicitly refer to them. (For more information, see Stoljar 2024; Gómez-Vírseda et al. 2019; Lewis 2019; Christman 2004; Khader 2020).

2 To avoid any possible misunderstanding, it should be emphasized at this point that individual and relational ideas of autonomy are different concepts. Individualistic views of autonomy should therefore not be regarded as hierarchically superior. Rather, what is crucial is the general approach, how exactly autonomy is understood, and what influence a person’s social environment can have on this.

3 If the person with dementia is in a phase in which their own wishes, ideas about life and beliefs can no longer be communicated (e.g., due to a lack of mental capacity), they no longer meet the criteria of an overly individualistic concept of autonomy.

4 It should be noted, however, that the robotic seal Paro is one of the most widely used robots in the context of dementia. This is particularly due to the fact that the possibilities for interaction with this companion are relatively limited, which is well suited for people with dementia and their decreasing physical and mental abilities. Apart from that, however, this article examines socially assistive robots with more extensive technical possibilities in order to assess their potential for supporting people with dementia, which has not yet been fully exploited. (For more in-depth information, see, for example, Pfadenhauer and Dukat 2015; Ghafurian et al. 2021; Moyle et al. 2019).

5 This disadvantage relates to the dementia disease itself and illustrates the importance of an already established relationship. At the same time, however, the aspect of novelty is emphasized in this area. The only important fact in this context is that people with dementia forget certain memory contents due to their illness, but this has an apparently negligible relevance for AI-based robots. They cannot lack experience, so to speak, compared to those people who do not have dementia, because these people usually have no experience with robots either—the decisive factor is rather the positive effect of the *new.*

6 Regarding this technical relationship, the actor-network theory (ANT) can be meaningfully integrated, according to which non-human entities can also be regarded as actors. Such entities (e.g., care robots) are integrated into a social structure and fulfill a specific role in the social world. Authors emphasizes the possibility of a relationship with robots—as artificial actors. The robot appearance, or physical presence, is extremely important for many people in need of care, but it does not allow for general conclusions (Coeckelbergh 2011; Cresswell et al. 2010; Elder-Vass 2015; Kropf 2025).

7 The decision-making process of AI systems, which is becoming increasingly difficult for experts to comprehend, is often described as a black-box. The sensory inputs are stored as data and then converted into an output with the help of algorithms. The steps between input and output sometimes take place in hidden layers, which, due to their complexity, are increasingly difficult to understand and control. However, it can stimulate reflection that human intuition can also be seen as a black-box (Bartneck et al. 2021; Gille et al. 2020).

8 Some studies show that personalizing a machine is of crucial importance. In the case of people with dementia, it is not only about recognizing specific needs, focusing on exactly this one person, and providing individual suggestions, but also about meeting the cognitive stage. Designing care robots in this way does not yet seem fully feasible, but it should be seen as a long-term goal. See, for example, (Law et al. 2019; Roger et al. 2012; Robaczewski et al. 2021; Sorell and Draper 2014; Chita-Tegmark and Scheutz 2021; Kropf 2025).

9 The authors argue that monitoring the elderly population with robots carries the risk that this may feel like being in prison, where the people concerned feel observed and controlled. However, based on the arguments so far, this risk can also be considered real when human nursing staff are involved (Sharkey and Sharkey 2012, 33).

## References

[CR1] Alzheimer’s Disease International. 2025. Dementia Statistics. https://www.alzint.org/about/dementia-facts-figures/dementia-statistics/. Accessed 11 Feb 2025.

[CR2] Anderson, Joel. 2013. Relationale Autonomie 2.0. In *Patientenautonomie: Theoretische Grundlagen – Praktische Anwendungen*, eds. Claudia Wiesemann and Alfred Simon, 61–75. Paderborn: Mentis Verlag.

[CR3] Banovic, Silva, Lejla Junuzovic Zunic, and Osman Sinanovic. 2018. Communication Difficulties as a Result of Dementia. *Materia Socio-Medica* 30 (3): 221–224. 10.5455/msm.2018.30.221-224. 30515063 10.5455/msm.2018.30.221-224 PMC6195406

[CR4] Bartneck, Christoph, Christoph Lütge, Alan Wagner, and Sean Welsh. 2021. *An Introduction to Ethics in Robotics and AI*. Cham: Springer International Publishing.

[CR5] Beauchamp, Tom L., and James F. Childress. 2019. *Principles of Biomedical Ethics*, 8th ed. New York: Oxford University Press.

[CR6] Bender, Andreas. 2018. Demenz-Syndrome. In *Kurzlehrbuch Neurologie*, eds. Andreas Bender, Jan Rémi, Berend Feddersen, et al. 3rd edn, 275–86. Kurzlehrbuch: Elsevier.

[CR7] Blankevoort, Christiaan G., Marieke J. G. van Heuvelen, Froukje Boersma, Helga Luning, Jeltsje de Jong, and Erik J. A. Scherder. 2010. Review of Effects of Physical Activity on Strength, Balance, Mobility and ADL Performance in Elderly Subjects with Dementia. *Dementia and Geriatric Cognitive Disorders* 30 (5): 392–402. 10.1159/000321357. 20980758 10.1159/000321357

[CR8] Buchs, Stefan. 2018. *Ärzteethos und Suizidbeihilfe: Theologisch-ethische Untersuchung zur Praxis der ärztlichen Suizidbeihilfe in der Schweiz.* Studien zur theologischen Ethik v.151. Basel: Schwabe Verlag Basel. https://ebookcentral.proquest.com/lib/kxp/detail.action?docID=5485496. Accessed 5 Nov 2025.

[CR9] Chan, Gary Kok Yew. 2021. Trust in and Ethical Design of Carebots: The case for ethics of care. *International Jornal of Social Robotics* 13 (4): 629–645. 10.1007/s12369-020-00653-w. 10.1007/s12369-020-00653-w PMC7245509 32837630

[CR10] Chita-Tegmark, Meia, and Matthias Scheutz. 2021. Assistive Robots for the social management of health: a framework for robot design and human-robot interaction research. *Intenational Journal of Social Robotics* 13 (2): 197–217. 10.1007/s12369-020-00634-z. 10.1007/s12369-020-00634-z PMC7223628 32421077

[CR11] Christman, John. 2004. Relational autonomy, liberal individualism, and the social constitution of selves. *Philosophical Studies: An International Journal for Philosophy in the Analytic Tradition* 117 (1/2): 143–64. http://www.jstor.org/stable/4321441.

[CR12] Christman, John. 2014. Relational autonomy and the social dynamics of paternalism. *Ethical Theory and Moral Practice* 17 (3): 369–382. 10.1007/s10677-013-9449-9.

[CR13] Christman, John, and Joel Anderson, eds. 2005. *Autonomy and the Challenges to Liberalism: New **Essays*, 1st edition. Cambridge: Cambridge University Press.

[CR14] Chu, Mei-Tai., Rajiv Khosla, Seyed Mohammad Sadegh. Khaksar, and Khanh Nguyen. 2017. Service innovation through social robot engagement to improve dementia care quality. *Assistive Technology *29 (1): 8–18. 10.1080/10400435.2016.1171807. 10.1080/10400435.2016.1171807 27064692

[CR15] Coeckelbergh, Mark. 2011. Humans, animals, and robots: a phenomenological approach to human-robot relations. *International Journal of Social Robotics* 3 (2): 197–204. 10.1007/s12369-010-0075-6.

[CR16] Coeckelbergh, Mark. 2012. Can We Trust Robots? *Ethics and Information Technology* 14 (1): 53–60. 10.1007/s10676-011-9279-1.

[CR17] Cresswell, Kathrin M., Allison Worth, and Aziz Sheikh. 2010. Actor-Network Theory and Its Role in Understanding the Implementation of Information Technology Developments in Healthcare. *BMC Medical Informatics and Decision Making* 10 (67): 1–11. 10.1186/1472-6947-10-67. 21040575 10.1186/1472-6947-10-67 PMC2988706

[CR18] Dosso, Jill A., Ela Bandari, Aarti Malhotra, et al. 2022. User perspectives on emotionally aligned social robots for older adults and persons living with dementia. *Journal of Rehabilitation and Assistive Technologies Engineering* 9 (1): 20556683221108364. 10.1177/20556683221108364. 10.1177/20556683221108364 PMC9248047 35782883

[CR19] Dove, Edward S., Susan E. Kelly, Federica Lucivero, Mavis Machirori, Sandi Dheensa, and Barbara Prainsack. 2017. Beyond Individualism: Is There a Place for Relational Autonomy in Clinical Practice and Research? *Clinical Ethics* 12 (3): 150–165. 10.1177/1477750917704156. 28989327 10.1177/1477750917704156 PMC5603969

[CR20] Draper, Heather, and Tom Sorell. 2017. Ethical Values and Social Care Robots for Older People: An International Qualitative Study. *Ethics and Information Technology* 19 (1): 49–68. 10.1007/s10676-016-9413-1.

[CR21] Ebert, Alexandria R., Danica Kulibert, and Susan H. McFadden. 2020. Effects of dementia knowledge and dementia fear on comfort with people having dementia: Implications for Dementia-Friendly Communities. *Dementia* 19 (8): 2542–2554. 10.1177/1471301219827708. 10.1177/1471301219827708 30739490

[CR22] Elder-Vass, Dave. 2015. Disassembling Actor-Network Theory. *Philosophy of the Social Sciences* 45 (1): 100–121. 10.1177/0048393114525858.

[CR23] Frankfurt, Harry G. 1988. Freedom of the Will and the Concept of a Person. In *What Is a Person?*, ed. Michael F. Goodman, 127–144. Totowa: Humana Press.

[CR24] Ghafurian, Moojan, Jesse Hoey, and Kerstin Dautenhahn. 2021. Social Robots for the Care of Persons with Dementia. *ACM Transactions on Human-Robot Interaction* 10 (4): 1–31. 10.1145/3469653.

[CR25] Gille, Felix, Anna Jobin, and Marcello Ienca. 2020. What We Talk About When We Talk About Trust: Theory of Trust for AI in Healthcare. *Intelligence-Based Medicine* 1–2: 100001. 10.1016/j.ibmed.2020.100001.

[CR26] Gómez-Vírseda, Carlos, Yves de Maeseneer, and Chris Gastmans. 2019. Relational Autonomy: what does it mean and how is it used in end-of-life care? A systematic review of argument-based ethics literature. *BMC Medical Ethics* 20: 76. 10.1186/s12910-019-0417-3. 10.1186/s12910-019-0417-3 PMC6815421 31655573

[CR27] Gómez-Vírseda, Carlos, Yves de Maeseneer, and Chris Gastmans. 2020. Relational autonomy in end-of-life care ethics: A contextualized approach to real-life complexities. *BMC Medical Ethics* 21: 50. 10.1186/s12910-020-00495-1. 10.1186/s12910-020-00495-1 PMC7325052 32605569

[CR28] Herrmann, Lynn K., Elisabeth Welter, James Leverenz, Alan J. Lerner, Nancy Udelson, Cheryl Kanetsky, and Martha Sajatovic. 2018. A systematic review of dementia-related stigma research: can we move the stigma dial? *American Journal of Geriatric Psychiatry *26 (3): 316–331. 10.1016/j.jagp.2017.09.006. 10.1016/j.jagp.2017.09.006 29426607

[CR29] Honekamp, Ivonne, Larissa Sauer, Thomas Wache, and Wilfried Honekamp. 2019. Akzeptanz Von Pflegerobotern Im Krankenhaus. *TATuP* 28 (2): 58–63. 10.14512/tatup.28.2.s58.

[CR30] Horn, Christoph, Corinna Mieth, and Nico Scarano. 2015. *Immanuel Kant Grundlegung zur Metaphysik der Sitten: Kommentar von Christoph Horn, Corinna Mieth und Nico Scarano*, 4rd edition. Frankfurt am Main: Suhrkamp.

[CR31] Hung, Lillian, Jim Mann, Jennifer Perry, Annette Berndt, and Joey Wong. 2022. Technological Risks and Ethical Implications of Using Robots in Long-Term Care. *Journal of Rehabilitation and Assistive Technologies Engineering* 9 (1): 20556683221106916. 10.1177/20556683221106917. 35733613 10.1177/20556683221106917 PMC9208036

[CR32] Hunt, Matthew R., and Carolyn Ells. 2011. Partners Towards Autonomy: Risky Choices and Relational Autonomy in Rehabilitation Care. *Disability and Rehabilitation* 33 (11): 961–967. 10.3109/09638288.2010.515703. 20822473 10.3109/09638288.2010.515703

[CR33] Ienca, Marcello, Fabrice Jotterand, Constantin Vică, and Bernice Elger. 2016. Social and assistive robotics in dementia care: Ethical recommendations for research and practice. *International Journal of Social Robotics* 8 (4): 565–573. 10.1007/s12369-016-0366-7.

[CR34] iTMunch. 2019. Robotics for more than just automation: Robots to help patients with dementia and alzheimer’s. https://itmunch.com/automation-robot-help-patients-dementia-and-alzheimers/. Accessed 20 February 2025.

[CR35] Johnston, Carolyn. 2022. Ethical Design and Use of Robotic Care of the Elderly. *Journal of Bioethical Inquiry* 19 (1): 11–14. 10.1007/s11673-022-10181-z. 35312965 10.1007/s11673-022-10181-z PMC8936033

[CR36] Khader, Serene J. 2020. The Feminist Case Against Relational Autonomy. *Journal of Moral Philosophy* 17 (5): 499–526. 10.1163/17455243-20203085.

[CR37] Koh, Wei Qi, Tijs Vandemeulebroucke, Chris Gastmans, Rose Miranda, and Lieve van den Block. 2023. The Ethics of Pet Robots in Dementia Care Settings: Care Professionals’ and Organisational Leaders’ Ethical Intuitions. *Frontiers in Psychiatry* 14: 1052889. 10.3389/fpsyt.2023.1052889. 36756218 10.3389/fpsyt.2023.1052889 PMC9899814

[CR38] Kropf, Mario. 2024. Spiritualität Im Kontext Von Demenzerkrankungen: Ethische Anforderungen Für Eine Ganzheitliche Betreuung Am Lebensende. *Spiritual Care* 13 (1): 33–41. 10.1515/spircare-2022-0085.

[CR39] Kropf, Mario. 2025. Trust and Care Robots: Philosophical Considerations, Ethical Challenges, and Viable Options. *Intelligent Service Robotics*, 5 February 2025. 10.1007/s11370-025-00589-y.

[CR40] Lam, Freddy M.H., Mei-Zhen Huang, Lin-Rong Liao, Raymond C.K. Chung, Timothy C.Y. Kwok, and Marco Y.C. Pang. 2018. Physical exercise improves strength, balance, mobility, and endurance in people with cognitive impairment and dementia: A Systematic Review. *Journal of Physiotherapy* 64 (1): 4–15. 10.1016/j.jphys.2017.12.001. 10.1016/j.jphys.2017.12.001 29289581

[CR41] Law, Mikaela, Craig Sutherland, Ho Seok Ahn, et al. 2019. Developing assistive robots for people with mild cognitive impairment and mild dementia: A qualitative study with older adults and experts in aged care. *British Medical Journal Open* 9: e031937. 10.1136/bmjopen-2019-031937. 10.1136/bmjopen-2019-031937 PMC6773341 31551392

[CR42] Le, Thi Dung, Shih-Chun. Lin, Mei-Chih. Huang, Sheng-Yu. Fan, and Chi-Yin. Kao. 2024. Factors Impacting the Demonstration of Relational Autonomy in Medical Decision-Making: A Meta-Synthesis. *Nursing Ethics* 31 (5): 714–738. 10.1177/09697330231200570. 37818823 10.1177/09697330231200570

[CR43] Leung, Mei-yung, Chendi Wang, and Timothy C. Y. Kwok. 2020. Effects of Supporting Facilities on Memory Loss Among Older People with Dementia in Care and Attention Homes. *Indoor and Built Environment* 29 (3): 438–448. 10.1177/1420326X19886344.

[CR44] Lewis, Jonathan. 2019. Does Shared Decision Making Respect a Patient’s Relational Autonomy? *Journal of Evaluation in Clinical Practice* 25 (6): 1063–1069. 10.1111/jep.13185. 31148321 10.1111/jep.13185

[CR45] Low, Lee-Fay., and Farah Purwaningrum. 2020. Negative stereotypes, fear and social distance: A systematic review of depictions of dementia in popular culture in the context of stigma. *BMC Geriatrics* 20: 477. 10.1186/s12877-020-01754-x. 10.1186/s12877-020-01754-x PMC7670593 33203379

[CR46] Mackenzie, Catriona. 2019a. Feminist Innovation in Philosophy: Relational Autonomy and Social Justice. *Women’s Studies International Forum* 72 (1): 144–151. 10.1016/j.wsif.2018.05.003.

[CR47] Mackenzie, Catriona. 2019. Relational Autonomy: State of the Art Debate. In *Spinoza and Relational Autonomy: Being with Others*, eds. Aurelia Armstrong, Keith Green, and Andrea Sangiacomo, 10–32. Edinburgh: Edinburgh University Press.

[CR48] Mackenzie, Catriona, and Natalie Stoljar. 2000. Introduction: Autonomy Refigured. In *Relational Autonomy: Feminist Perspectives on Automony, Agency, and the Social Self*, ed. Catriona Mackenzie and Natalie Stoljar, 3–34. Oxford: Oxford University Press Incorporated.

[CR49] Mannion, Arlene, Sarah Summerville, Eva Barrett, et al. 2020. Introducing the social robot mario to people living with dementia in long term residential care: Reflections. *Internatioal Journal of Social Robotics* 12 (2): 535–547. 10.1007/s12369-019-00568-1.

[CR50] Menke, Christoph. 2010. Autonomie Und Befreiung. *Deutsche Zeitschrift Für Philosophie* 58 (5): 675–695. 10.1524/dzph.2010.58.5.675.

[CR51] Misztal, Barbara A. 2012. Trust: acceptance Of, arecaution against and cause of vulnerability. In *Trust: Comparative Perspectives*, eds. Masamichi S. Sasaki and Robert M. Marsh, 209–36. Leiden, Boston: Brill.

[CR52] Moyle, Wendy, Cindy Jones, Jenny Murfield, Lukman Thalib, Elizabeth Beattie, David Shum, and Brian Draper. 2019. Using a Therapeutic Companion Robot for Dementia Symptoms in Long-Term Care: Reflections from a Cluster-RCT. *Aging & Mental Health* 23 (3): 329–336. 10.1080/13607863.2017.1421617. 29282989 10.1080/13607863.2017.1421617

[CR53] Nakamura, Yoshiki, and Hiroyuki Umemuro. 2022. Effect of robot’s listening attitude change on self-disclosure of the elderly. *International Journal of Social Robotics* 14 (9): 1935–1950. 10.1007/s12369-022-00934-6. 10.1007/s12369-022-00934-6 PMC9553638 36247492

[CR54] Naneva, Stanislava, Marina Sarda Gou, Thomas L. Webb, and Tony J. Prescott. 2020. A systematic review of attitudes, anxiety, acceptance, and trust towards social robots. *International Journal of Social Robotics* 12 (6): 1179–1201. 10.1007/s12369-020-00659-4.

[CR55] Nebel, Rebecca A., Neelum T. Aggarwal, Lisa L. Barnes, et al. 2018. Understanding the Impact of Sex and Gender in Alzheimer’s Disease: A Call to Action. *Alzheimer’s & Dementia* 14 (9): 1171–1183. 10.1016/j.jalz.2018.04.008. 10.1016/j.jalz.2018.04.008 PMC6400070 29907423

[CR56] Nijssen, Sari R.R., Barbara C.N. Müller, Tibor Bosse, and Markus Paulus. 2022. Can You count on a calculator? The role of agency and affect in judgments of robots as moral agents. *Human-Computer Interaction* 38 (5-6): 400-416. 10.1080/07370024.2022.2080552.

[CR57] Obayashi, Kazuko, Naonori Kodate, and Shigeru Masuyama. 2020. Measuring the Impact of Age, Gender and Dementia on Communication-Robot Interventions in Residential Care Homes. *Geriatrics & Gerontology International* 20 (4): 373–378. 10.1111/ggi.13890. 32077237 10.1111/ggi.13890

[CR58] O’Brolcháin, Fiachra. 2019. Robots and People with Dementia: Unintended Consequences and Moral Hazard. *Nursing Ethics* 26 (4): 962–972. 10.1177/0969733017742960. 29262739 10.1177/0969733017742960

[CR59] Oksanen, Atte, Nina Savela, Rita Latikka, and Aki Koivula. 2020. Trust Toward Robots and Artificial Intelligence: An Experimental Approach to Human-Technology Interactions Online. *Frontiers in Psychology* 11: 568256. 10.3389/fpsyg.2020.568256. 33343447 10.3389/fpsyg.2020.568256 PMC7744307

[CR60] Oshana, Marina A.L. 2005. Autonomy and Self-Identity. In *Autonomy and the Challenges to Liberalism: New Essays*, 1st edition, edited by John Christman and Joel Anderson, 77–100. Cambridge: Cambridge University Press.

[CR61] Oshana, Marina A.L. 2013. Relational Autonomy. In *The International Encyclopedia of Ethics*, ed. Hugh LaFollette, 1–13. Chichester: Wiley-Blackwell.

[CR62] Persson, Marcus, Clara Iversen, and David Redmalm. 2024. Making Robots Matter in Dementia Care: Conceptualising the Triadic Interaction Between Caregiver, Resident and Robot Animal. *Sociology of Health & Illness* 46 (6): 1192–1211. 10.1111/1467-9566.13786. 38733615 10.1111/1467-9566.13786

[CR63] Pfadenhauer, Michaela, and Christoph Dukat. 2015. Robot caregiver or robot-supported caregiving? *Internaional Journal of Social Robotics* 7 (3): 393–406. 10.1007/s12369-015-0284-0.

[CR64] Poulsen, Adam, Oliver K. Burmeister, and David Kreps. 2018. The ethics of inherent trust in care robots for the elderly. In *This Changes Everything – ICT and Climate Change: What Can We Do?* Vol. 537, eds. David Kreps, Charles Ess, Louise Leenen, and Kai Kimppa, 314–28. IFIP Advances in Information and Communication Technology. Cham: Springer International Publishing.

[CR65] Robaczewski, Adam, Julie Bouchard, Kevin Bouchard, and Sébastien. Gaboury. 2021. Socially assistive robots: The specific case of the nao. *International Journal of Social Robotics* 13 (4): 795–831. 10.1007/s12369-020-00664-7.

[CR66] Roger, Kerstin, Lorna Guse, Elaine Mordoch, and Angela Osterreicher. 2012. Social Commitment Robots and Dementia. *Canadian journal on aging *31 (1): 87–94. 10.1017/S0714980811000663. 10.1017/S0714980811000663 22336517

[CR67] Ryan, Mark. 2020. In AI We Trust: Ethics, Artificial Intelligence, and Reliability. *Science and Engineering Ethics* 26 (5): 2749–2767. 10.1007/s11948-020-00228-y. 32524425 10.1007/s11948-020-00228-y PMC7550313

[CR68] Sharkey, Amanda, and Noel Sharkey. 2012. Granny and the Robots: Ethical Issues in Robot Care for the Elderly. *Ethics and Information Technology* 14 (1): 27–40. 10.1007/s10676-010-9234-6.

[CR69] Soljacic, Fran, Theresa Law, Meia Chita-Tegmark, and Matthias Scheutz. 2024. Robots in healthcare as envisioned by care professionals. *Intelligent Service Robotics* 17 (3): 685–701. 10.1007/s11370-024-00523-8.

[CR70] Sorell, Tom, and Heather Draper. 2014. Robot Carers, Ethics, and Older People. *Ethics and Information Technology* 16 (3): 183–195. 10.1007/s10676-014-9344-7.

[CR71] Stoljar, Natalie. 2024. Feminist Perspectives on Autonomy. In *The Stanford Encyclopedia of Philosophy (Summer 2024 Edition)*, eds. Edward N. Zalta and Uri Nodelman. Stanford: Metaphysics Research Lab, Stanford University.

[CR72] Specker Sullivan, Laura, and Fay Niker. 2018. Relational Autonomy, Paternalism, and Maternalism. *Ethical Theory and Moral Practice* 21 (3): 649–667. http://www.jstor.org/stable/44955657.

[CR73] Sweeney, Paula. 2023. Trusting Social Robots. *AI and Ethics* 3 (2): 419–426. 10.1007/s43681-022-00165-5. 35634257 10.1007/s43681-022-00165-5 PMC9127473

[CR74] Tampi, Rajesh R., and Dilip V. Jeste. 2022. Dementia is more than memory loss: Neuropsychiatric Symptoms of dementia and their nonpharmacological and pharmacological management. *American Journal of Psychiatry* 179 (8): 528–543. 10.1176/appi.ajp.20220508. 10.1176/appi.ajp.20220508 35921394

[CR75] Tamura, Toshiyo, Satomi Yonemitsu, Akiko Itoh, et al. 2004. Is an Entertainment Robot Useful in the Care of Elderly People with Severe Dementia? *Journals of Gerontology:* *Series A* 59 (1): M83–M85. 10.1093/gerona/59.1.M83. 10.1093/gerona/59.1.m83 14718491

[CR76] Tanioka, Tetsuya, Tomoya Yokotani, Ryuichi Tanioka, et al. 2021. Development Issues of healthcare robots: Compassionate communication for older adults with dementia. *International Journal of Environmental Research and Public Health* 18 (9): 4538. 10.3390/ijerph18094538. 10.3390/ijerph18094538 PMC8123161 33923353

[CR77] Thompson, Christopher. 2017. Trust Without Reliance. *Ethical Theory and Moral Practice* 20 (3): 643–55. http://www.jstor.org/stable/44955541.

[CR78] Trahan, Maranda A., SungWoo Kahng, Alyssa B. Fisher, and Nicole L. Hausman. 2011. Behavior-Analytic Research on Dementia in Older Adults. *Journal of Applied Behavior Analysis* 44 (3): 687–691. 10.1901/jaba.2011.44-687. 21941406 10.1901/jaba.2011.44-687 PMC3177357

[CR79] Vandemeulebroucke, Tijs, Kevin Dzi, and Chris Gastmans. 2021. Older Adults’ experiences with and perceptions of the use of socially assistive robots in aged care: A systematic review of quantitative evidence. *Archives of Gerontology and Geriatrics* 95: 104399. 10.1016/j.archger.2021.104399. 10.1016/j.archger.2021.104399 33813208

[CR80] Vanlaere, Linus, and Chris Gastmans. 2011. A Personalist Approach to Care Ethics. *Nursing Ethics* 18 (2): 161–173. 10.1177/0969733010388924. 21372229 10.1177/0969733010388924

[CR81] Walter, Jennifer K., and Lainie Friedman Ross. 2013. Relational Autonomy as the key to effective behavioral change. *Philosophy, Psychiatry, & Psychology* 20 (2): 169–77. 10.1353/ppp.2013.0028.

[CR82] Williams, Kristine N., Ruth Herman, Byron Gajewski, and Kristel Wilson. 2009. Elderspeak Communication: Impact on Dementia Care. *American Journal of Alzheimer’s Disease and Other Dementias* 24 (1): 11–20. 10.1177/1533317508318472. 10.1177/1533317508318472 PMC2823803 18591210

[CR83] Yuan, Fengpei, Joel G. Anderson, Tami H. Wyatt, Ruth Palan Lopez, Monica Crane, Austin Montgomery, and Xiaopeng Zhao. 2022. Assessing the acceptability of a humanoid robot for alzheimer’s disease and related dementia care using an online survey. *Internationa Journal of Social Robotics* 14 (5): 1223–1237. 10.1007/s12369-021-00862-x.

[CR84] Zuschnegg, Julia, Lucas Paletta, Maria Fellner, et al. 2022. Humanoid socially assistive robots in dementia care: a qualitative study about expectations of caregivers and dementia trainers. *Aging & Mental Health* 26 (6): 1270–1280. 10.1080/13607863.2021.1913476. 10.1080/13607863.2021.1913476 33904791

